# Pandemic infection rates are deterministic but cannot be modeled

**DOI:** 10.1063/5.0015303

**Published:** 2020-11-24

**Authors:** Joseph L. McCauley

**Affiliations:** Physics Department, University of Houston, Houston, Texas 77204, USA

## Abstract

The covid-19 infection rates for a large number of infections collected from a large number of different sites are well defined with a negligible scatter. The simplest invertible iterated map, exponential growth and decay, emerges from country-wide histograms whenever Tchebychev’s inequality is satisfied to within several decimal places. This is one point. Another is that failed covid-19 pandemic model predictions have been reported repeatedly by the news media. Model predictions fail because the observed infection rates are beyond modeling: any model that uses fixed rates or uses memory or averages of past rates cannot reproduce the data on active infections. When those possibilities are ruled out, then little is left. Under lockdown and social distancing, the rates unfold daily in small but unforeseeable steps, they are algorithmically complex. We can, however, use two days in the daily data, today and any single day in the past (generally yesterday), to make a useful forecast of future infections. No model provides results better than this simple forecast. We analyze the actual doubling times for covid-19 data and compare them with our predicted doubling times. Flattening and peaking are precisely defined. We identify and study the separate effects of social distancing vs recoveries in the daily infection rates. Social distancing can only cause flattening but recoveries are required in order for the active infections to peak and decay. Three models and their predictions are analyzed. Pandemic data for Austria, Germany, Italy, the USA, the UK, Finland, China, Taiwan, and Sweden are discussed.

## I. EXPONENTIAL ITERATED MAPS AND THE PANDEMIC DATA

**FIG. 1.**
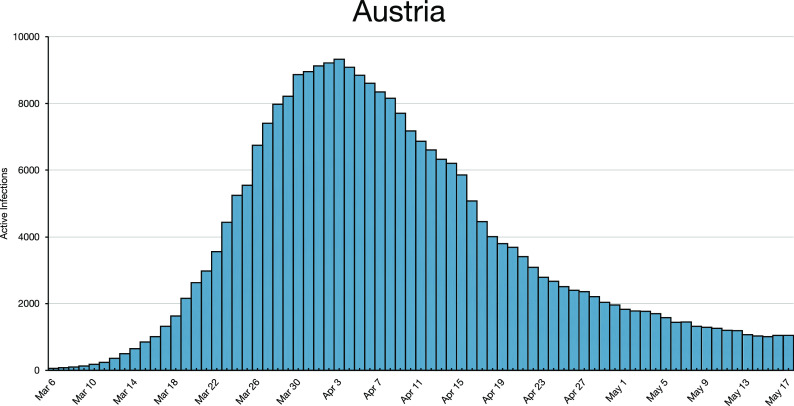
Austria (with a population of 8 × 10^6^, 1/10th of Germany) peaked on April 3, 2020 and reopened small businesses on April 14, 2020. On June 15, 2020, when the borders reopened, masks were no longer required in businesses. It took 2.5 weeks from lockdown to peak and then 5.5 weeks to decay to the infection level of March 16, 2020 (lockdown).

**FIG. 2.**
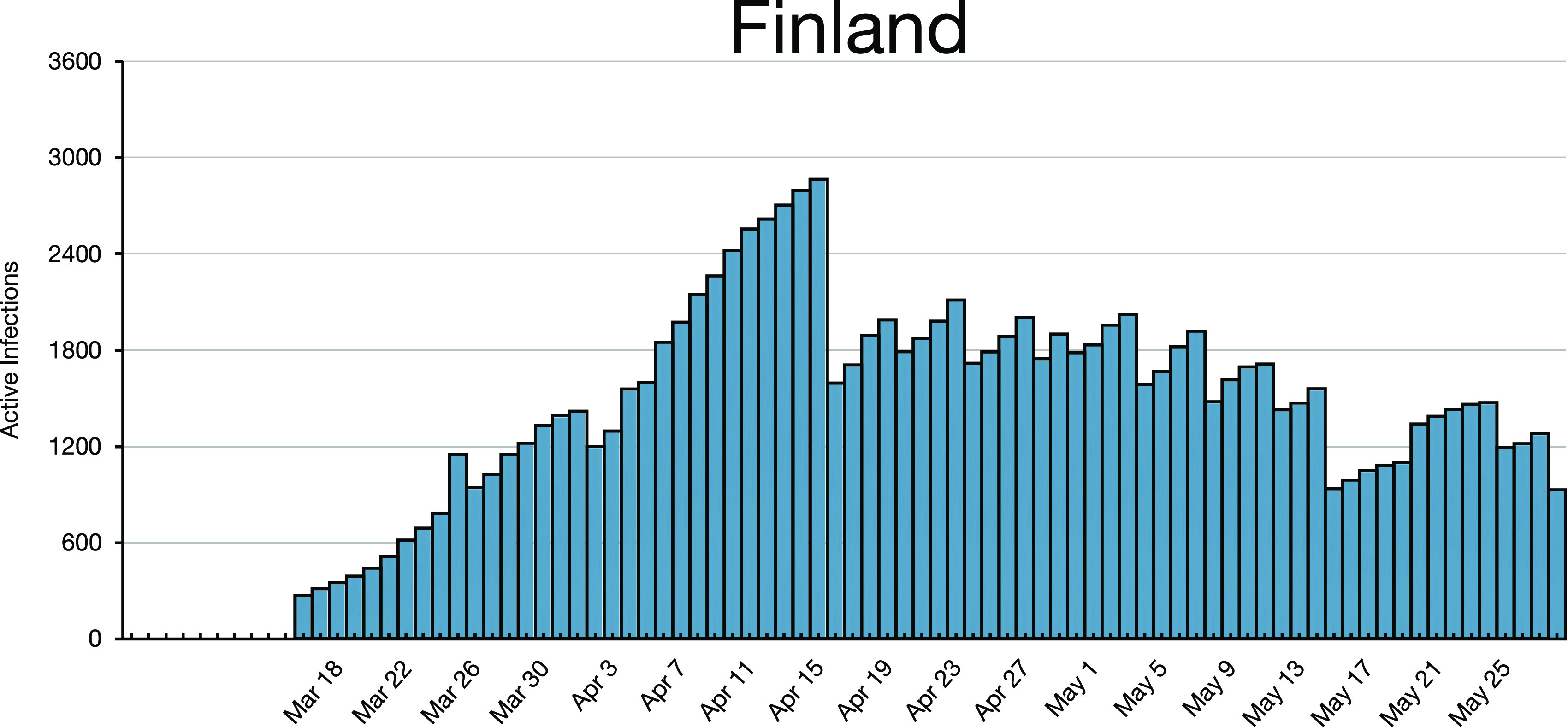
Finland’s oscillating active infections after peaking on April 1, 2020. Finland’s population is less than 3/4 that of Austria.

**FIG. 3.**
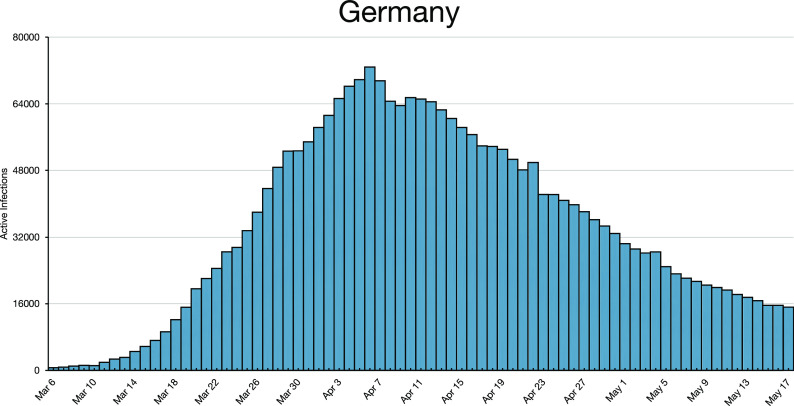
Germany peaked on April 6, 2020 and partly reopened in April. The border with Austria remained closed except for work commuting. The borders reopened on June 15, 2020 but masks were still worn in German businesses into July. Germany did not decay to the level of March 16, 2020 until June 10, 2020, nearly three months later.

**FIG. 4.**
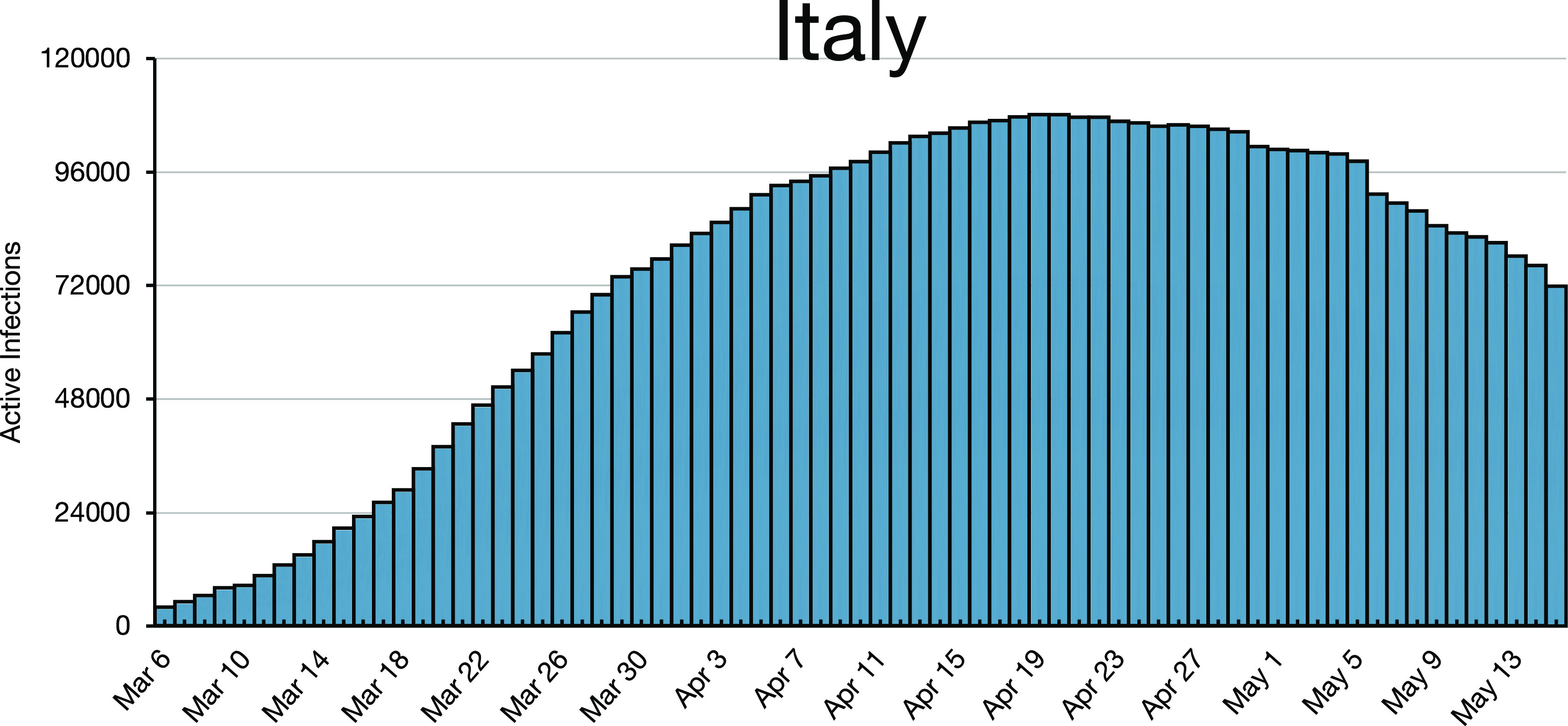
Italy (with a population 3/4 that of Germany and the same as that of the UK) flattened for 3 weeks without peaking, then peaked on April 20, 2020 with *r* ≈ 1.004.

**FIG. 5.**
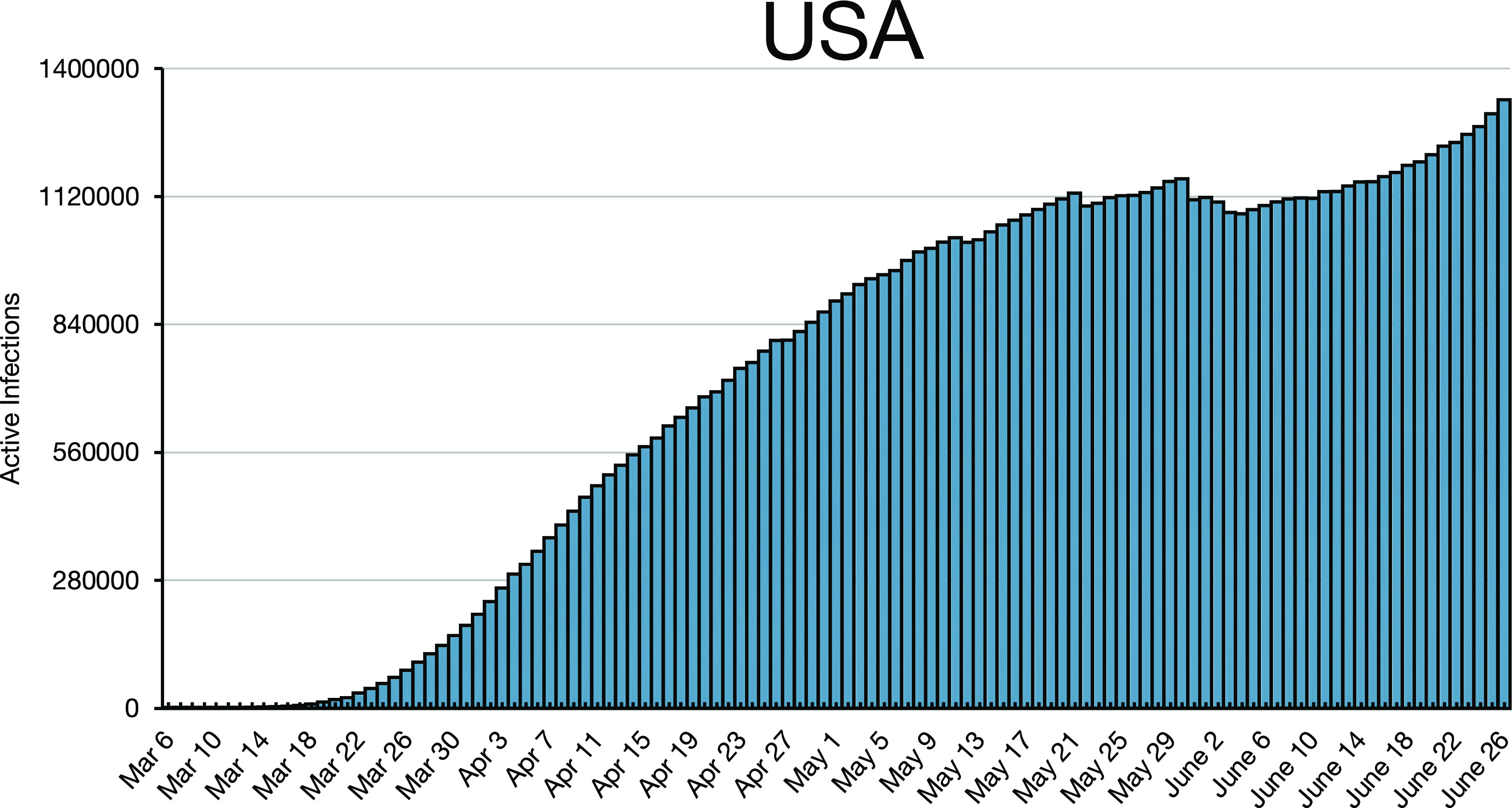
Well over two months after Austria and Germany peaked, the US infections were still climbing, first due to inadequate lockdown and social distancing, and then in June due to reopening, despite still growing infections. The US population is slightly over four times that of Germany.

**FIG. 6.**
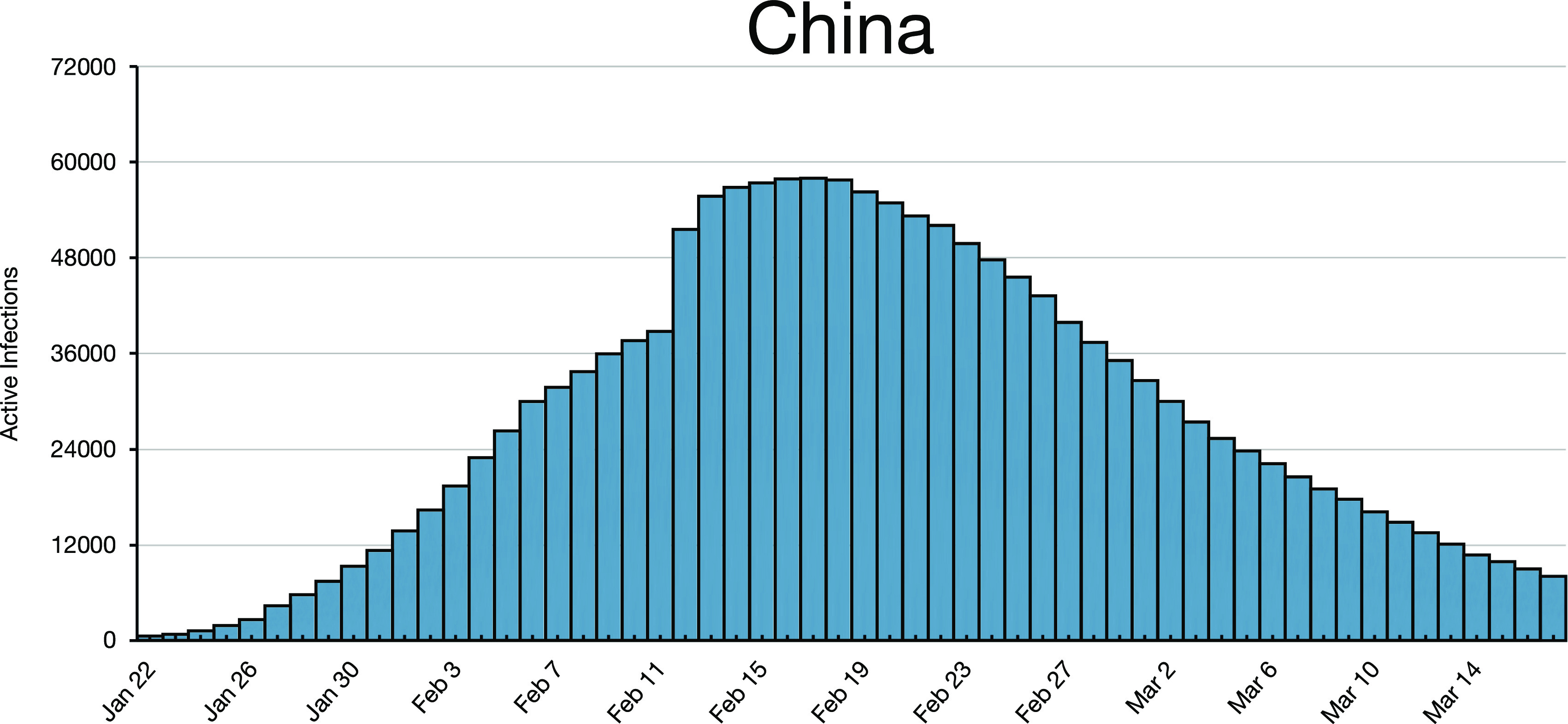
The data from China. This histogram is smooth compared with Figs. 1–5, especially for Germany where the numbers of active infections are of the same order of magnitude as for China.

**FIG. 7.**
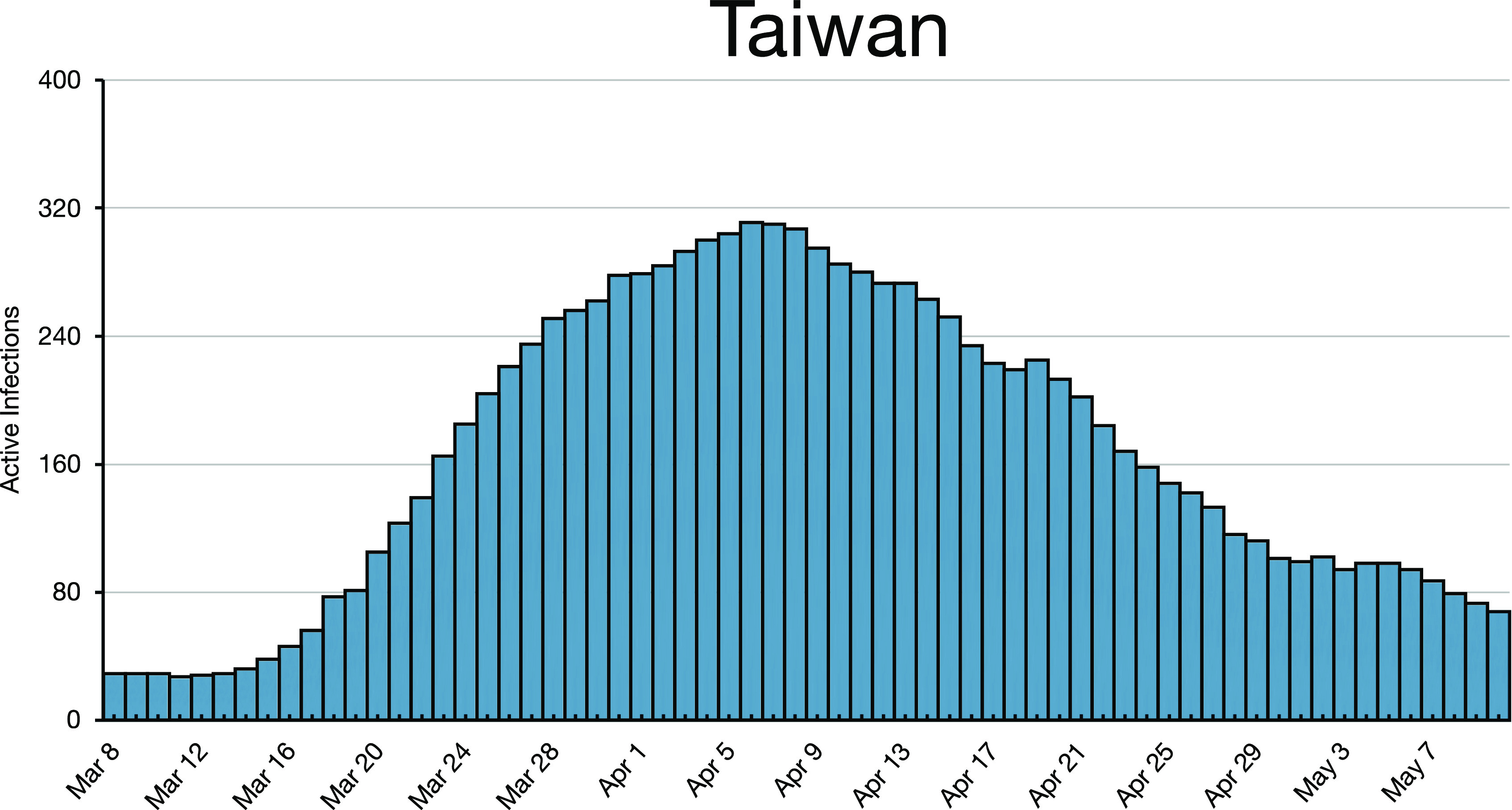
Taiwan peaked at about the same time as Austria and Germany.

The pandemic infection rates to two to three decimal places are an example of complexity: the infection rates can be calculated daily from the infections but they cannot be modeled and predicted from fixed initial conditions. Averaging past rates, or taking past rates as initial conditions, cannot tell us future rates. Any attempt to fix the infection and/or death rates in advance in order to make predictions from a model will be defeated by the daily unfolding. You can watch the unfolding, you can take measures to reduce infections, but you cannot predict when the active infections will flatten, or will peak and then decay, or if they will merely flatten without peaking. One cannot correctly predict a peak before it occurs. We state conditions for flattening/plateauing and also for peaking and decay in Sec. II. This is not about finding a formula or computer simulation to replace a discrete set of points with daily changing rates by a smooth curve. Such an effort would distract us from our job: nature has given us the data. Our job is to understand the data. This means that no arbitrary parameters will be introduced. The only quantities discussed below are determined daily by the data. Instead of fixing parameters in equations to try to predict the unpredictable, we will simply apply a good approximate number conservation law aided by kinetic equations to help us to understand the daily data. The histograms shown in Figs. 1–7 were constructed using worldometer data.^1^ We have noticed that worldometer sometimes updates their histograms; the active infections written down a week ago may be different today. As long as the differences are small this will not matter. In the cases of some countries not analyzed here there were enormous sudden jumps, as in the case of Norway on May 22 by a factor of more than 10^2^. Most likely, there were discoveries of discrepancies in reporting. In the case of the UK, the recoveries were always N/A on worldometer.

Simple exponential growth with a constant infection rate *r* is described by

$$I\left(n\right)=rI\left(n-1\right),$$ (1)

so that after *n* days, we would have a global infection rate

$$I\left(n\right)=r^{\mathrm{n-1}}I\left(1\right).$$ (2)

There is a bifurcation from growth to decay at *r* = 1; if *r* < 1, then the contagion dies out exponentially in the population. The class of map (1) describes the dynamics of an epidemic: the doubling time characterizes the global exponential growth. The doubling time would be *N* = ln 2/ln *r* if *r* > 0 were the daily rate constants. The best empirical test for exponential growth is that the data show a doubling time for each day.

We do not know how accurate are the data for a given country. We are concerned here with presenting a new method for understanding the data. The better the data, the better our understanding of an epidemic but the method will be the same in any case. It suffices to say that errors in reporting will be magnified at the rate λ = ln *r* if *r* > 1 and contracted at the rate λ = ln *r* if *r* < 1. Errors in data matter a lot during the growth of an epidemic but matter less as the epidemic dies out. Instead of (1), the infection rate varies daily with social distancing, *r*_*n*_ = *I*(*n*)/*I*(*n* − 1). Would infections be bounded (or occur in a circle), then we would have a Bernoulli shift. In our case, the exponential growth and decay of infections are described by local Liapunov exponents λ_*n*_ = ln *r*_*n*_,^2^ where *r*_*n*_ is the rate on day *n*. “Local” here means over short times and global means over long times (over many days or weeks) in the sense of mathematics, not geography.

The data are presented for each country by worldometer^2^ as finite size points that look superficially like a curve, but they are histograms with bins the width of one day as we have presented them in Figs. 1–7. Early in the pandemic with very few people infected, *I* ≈ 10–10^2^; with so little data, the infection rate *r* is erratic with much scatter due to too few points in the histograms. It makes no sense to try to construct an average of the infection rate *r* for times over which social distancing is in effect because, with social distancing, the rate *r* tends to fall systematically and it is the time change in a well-defined rate that interests us. The scatter in an ensemble average should also be great if we would take only a few collection sites (a few towns or districts) rather than many sites. When there are many more infections per day, *I* > 10^4^ for March 17, 2020 and beyond, then social distancing and lockdowns systematically reduce *r* daily, and it is that time dependence that we want to characterize and understand. In order to discover the natural rate of covid infection unchanged by restrictions on the freedom of movement and association, we analyze the data for Sweden in Sec. VI.

Infections spread from one person to another with some unknown local probability. The only possible basis for the exponential behavior of the infection rate must be Tchebychev’s inequality, the weak form of the law of large numbers.^3^ First, we need a statistical ensemble.^4^ Toward that end, consider one country in a *gedankendatensammlung* (gedanken data collection): inside the country, there are *M*_*n*_ different geographically nonoverlapping sites that report active infections on day *n*. A random variable r is defined as any variable that can be described by (or generates) a probability distribution, whether deterministically^1^ or stochastically.^4^ Let *P*(*r*) denote the empirical distribution (we use only the frequency definition of probability) for the random variable *r*_*n*_ = *I*(*n*)/*I*(*n* − 1), the infection rate. The number of infections *I*(*n* − 1) and *I*(*n*) on days *n* − 1 and *n*, respectively, are collected or recorded at *M*_*n*−1_ different sites. We then form the rate for day *n* − 1 from *I*(*n*)/*I*(*n* − 1), where *r*_*ni*_ = (*I*(*n*)/*I*(*n* − 1))_*i*_ for the ith region. The ensemble average rate for the entire land for day *n* − 1 to day *n* is then

$$r_{n}=\frac{1}{M_{n-1}}\sum_{i=1}^{M_{n-1}}{\left[\frac{I\left(n\right)}{I\left(n-1\right)}\right]}_{i}.$$ (3a)

At some stage of the infection, we will need to know the rates to within some numerical accuracy a, which we will specify below. Divide the *r*-axis into K bins, each of width a. The appropriate coarse-grained empirical probability is then

$$P\left(r\right)=\frac{1}{M_{n-1}}\sum_{k}^{K_{}}n_{k}\theta \left(r-r_{k}\right)$$ (3b)

with

$$r_{n}=\frac{1}{M_{n-1}}\sum_{k}^{K_{}}n_{k}r_{nk}.$$ (3c)

Equation (3c) is the same as Eq. (3a), where *n*_*k*_ is the number of times that the value *r*_*k*_ occurs in the *k*th bin. Our ensemble provides the complete statistical description of a pandemic or an epidemic. We next treat the infections from region to region as approximately statistically independent at the level of 1-point distribution in the hierarchy of distributions. The 2-point distribution^4^ would be required to study correlations from region to region. Tchebychev’s inequality^3^ then tells us that the ensemble average converges in probability to a well-defined rate *r*(*n*) to within accuracy a (the scatter is less than the bin size),

$$P\left(\left|\frac{1}{M_{n-1}}\sum_{i_{}}{\left[\frac{I\left(n\right)}{I\left(n-1\right)}\right]}_{i}-r\left(n\right)\right|\geq a\right)<\sigma^{2}\left(n\right)/M_{n}a^{2},$$ (4)

where σ^2^ is the mean square fluctuation calculated from *P*(*r*). For a = 10^−2^, with no help from the variance we would need M_n_ ≫ 10^4^ for the mean to dominate the scatter, but more generally we need σ^2^/*M*_*n*_ ≫ 10^−4^ for an accurate statistical ensemble average rate to reflect the pandemic data (we need a = 10^−3^ at late stages for Figs. 4 and 5, e.g.). As an example, in Pearson’s 20th century coin tossing experiment, the relative frequency of heads was n/M = 0.5016 for M = 12 000 tosses and n/M = 0.5005 for M = 24 000.^3^ Only the orders of magnitude matter (merely doubling M does not help much): with a = 10^−2^, σ ≈ 10^−1^ and M ≈ 10^4^, we get $P\left(\left|\frac{n}{M}-0.50\right|>10^{-2}\right)<10^{-1}$, or 10% chance of not hitting the mark in 12 000 tosses. Tchebychev’s inequality should provide the basis for the exponential decay of a radioactive element, where the decay rate is constant. In our case, Tchebychev’s inequality provides the condition for a well-defined infection rate, and therefore, the deterministic map from day *n* − 1 to day *n* emerges to within decimal accuracy from the underlying probabilistic nature of the spread of the disease at the microscale of one person to another. This knowledge does not allow us to predict *I*(*n* + 1), however, because the analysis for days *n* − 1 and *n* does not and cannot tell us *r*(*n* + 1). We would face the same unpredictability with coin tossing ensembles if, every day, we would be given a new coin with different and unknown moment of inertia. In that case, discovering the coin toss probability *p*(*n*) for day *n* would not give us any information about the probability *p*(*n* + 1) for day *n* + 1.

In what follows, we will simply write *r*(*n*) = *r*_*n*_. In some works, the difference Δ*I*(*n*) = *I*(*n* + 1) − *I*(*n*) is plotted but the statistically better behaved quantity to study is Δ*I*(*n*)/*I*(*n*) = *r*_*n*_ − 1, or more simply Eq. (5). With the daily rate *r*_*n*_ varying, we can then ask when the rate from day 0 to day *n* will be globally exponentially increasing. From

$$\frac{I\left(n\right)}{I\left(n-1\right)}=r_{n}$$ (5)

follows

$$I\left(n\right)=r_{n}\ldots r_{1}I\left(1\right)$$ (6)

so that the effective rate from day 0 to day *n* is given by

$$r^{n}\left(n,1\right)=r_{n}\ldots r_{1}$$ (7)

and λ_*n*_ = ln[*r*^n^(*n*, 1]) is the corresponding global Liapunov exponent. As a simple mathematical example, if *r*_*n*_ = 1 + ε/*n*, then the infections would tend to plateau forever as *n* increases. Plateauing for a long time without peaking is entirely possible, as is increasing without plateauing, and we will give examples in Sec. IV. *Plateauing, peaking, and decaying do not depend on initial conditions of active infections in the distant past, they depend only on social behavior and recoveries prior to a peak, if a peak would occur*. The usual division of the world (environment) into dynamical system plus initial conditions, in order to make global predictions from initial data, fails here.

*I*(*n*) describes the state of the dynamical system. The effective rate is not the rate on any given day, it is simply a number that directly connects two states of active infections on days 0 and *n*,

$$I\left(n\right)=r\left(n,1\right)I\left(1\right).$$ (8)

In other words, the states *I*(*n*) and *I*(*m*) are path-independent: any two states *I*(*n*) and *I*(*m*) are connected globally by a single exponential with the effective growth rate *r*(*n*, *m*) = *I*(*n*)/*I*(*m*). Furthermore, the sequence of steps does not matter, *I*(3)/*I*(1) = *r*_1_*r*_2_ = *r*_2_*r*_1_, so a drop in the rate following an increase, or vice-versa, is not significant for forecasting. If for each and every day we have *r*_*n*_ > 1, then *r*(*n*, 1) > 1, and the infections (6) are exponentially increasing. However, this condition is only sufficient and is not necessary: if *r*_*k*_ < 1 for some values of *k* = 1, …, *n* but the overall product (7) is greater than unity, then the process (8) is exponentially increasing through day *n*. Infections may decay, as in Austria after April 3, 2020 (Fig. 1), or they may oscillate, as for Finland after April 1, 2020 (Fig. 2), or they may increase with bumps and dips as in the case for the USA (Fig. 5). Any variation in daily slope that is above, equal to, or below unity is consistent with Eqs. (1) and (6). This is not an oversimplification: we are sticking to the data with accuracy to two to three decimal places.

We emphasize that Figs. 1 and 2 for Austria and Finland reflect the simple iterated map (5), respectively. You cannot generate the histograms from a stochastic process with noise the same order of magnitude as (or greater than) the drift (the observed infection rate). In contrast, a tiny (or undefinable) drift occurs for market data; market data are pure noise.^4^ In the language of a finance market, epidemic data for a large number of collection sites within a country reflect only the drift with a negligible scatter. A reader who doubts that the rates are well defined is invited to try constructing a stochastic dynamics that reproduces Figs. 1 and 2. It would make no sense to try constructing an ensemble using data from different countries because social distancing and recoveries vary from country to country. I have noted elsewhere that finance market indices do not meet the requirements for statistical ensembles.

Time reversibility is the signature of deterministic dynamics: the iterated map *I*(*n*) = *r*_*n*_*I*(*n* − 1) with *r*_*n*_ given by the data can be iterated either forward or backward in time *n* with the unique inverse *I*(*n* − 1) = *r*_*n*_^−1^*I*(*n*). Randomness (noise) at each time step would erase past, as in a finance market. It is impossible to describe stock, bond, or FX log returns via deterministic dynamics. Exponential growth is not only time reversible, the daily rates with 0 < *r*_*n*_ < ∞ form an Abelian group with the group multiplication rule (7). The infections on any two days *n* and *m* in the data are connected by an exponential with a single rate *r*(*n*, *m*). This is the group property (path independence of states).

## II. DOUBLING TIMES AND FORECASTING

The doubling time *N*_*n*_ with variable rate *r*_*n*_ is given by

$$I\left(n+N_{n}\right)=2I\left(n\right)$$ (9)

and depends on the starting day *n* (nonstationarity). A short doubling time provides the quickest test for exponential growth. Real doubling times may fall between two days so that *N*_*n*_ is generally not an integer. The observed doubling time starting from day *n* is then given in Tables I and II.

**TABLE I.** Observed doubling times for five countries calculated from our histograms. Germany, Austria, and the USA locked down on March 16, 2020, Italy on March 9, 2020, and the UK on March 23, 2020. Where a doubling time fell between 2 days, we labeled it as half a day. Before lockdown, the doubling time was 2–3 days. The doubling time reflects the effectiveness (or lack of same) of a lockdown. The USA and the UK lockdowns clearly were relatively ineffective. The data for the UK were later removed by worldometer.

Day *n*	Austria	Germany	Italy	UK	USA
March 16	3	2.5	6	3	2.5
March 17	3	2.5	6.5	3	1.5
March 18	3.5	4	7.5	3.5	2
March 19	3.5	5.5	8	4	2.5
March 20	4	6.5	11	4	2.5
March 21	4.5	6	14.5	4	2.5
March 22	4.5	6	15	4	3
March 23	7	9	20	3.5	3.5
March 24	>11	9	25	3.5	3.5
March 25	Peaked	9.5	Peaked	4.5	3.5
	April 3		April 21		
March 26		27		5	4.5
March 27		Peaked		5	5
		April 6			
March 28				5.5	5.5
March 29				5.5	5.5
March 30				6	5.5
March 31				6	6.5
April 1				7.5	8

**TABLE II.** Observed doubling time *N*_*n*_ vs the predicted doubling time *T*_*n*_ calculated from the daily infection rate for the UK and the USA. The time *N*_*n*_ is exact to within half a day, but when we approach the last date for which data are available, then *T*_*n*_ is the only estimate at our disposal.

Day *n*	UK *T*_*n*_	UK *N*_*n*_	USA *T*_*n*_	USA *N*_*n*_
March 16	3	3	2.5	2.5
March 17	2	3	1.5	1.5
March 18	1.5	3.5	2	2
March 19	3.5	4	2	2.5
March 20	3	4	3	2.5
March 21	6	4	2.5	2.5
March 22	4.5	4	3	3
March 23	3.5	3.5	3	3.5
March 24	4	3.5	3	3.5
March 25	3.5	4.5	3	3.5
March 26	3	5	3.5	4.5
March 27	4.5	5	4	5
March 28	5.5	5.5	5	5.5
March 29	5.5	5.5	5	5.5
March 30	6	6	5.5	5.5
March 31	5	7	5.5	6.5
April 1	4.6	7.5	5.5	7

We can also define a predicted doubling time (Tables II–VII) as

$$T_{n}=ln\;2/ln\;r_{n},$$ (10)

starting from day *n* where *r*_*n*_ = *I*(*n*)/*I*(*n* − 1). This will turn out to be a decent forecast of the future if and only if the daily rates do not deviate too much from *r*_*n*_ over the next *T*_*n*_ days. It is the only forecast that we will consider, although one can make a coarser-grained forecast by using two not-successive days. The predicted doubling time provides at least as good a forecast of the future as computer simulation models (I have checked this claim against many different model predictions of death rates in the USA). We can similarly write a predicted doubling time for the death rate, and the two rates are not the same. Deaths lag infections by about a month and are an order of magnitude smaller than active infections.

**TABLE III.** Austria’s daily growth rates *r* = *I*(*n* + 1)/*I*(*n*) read by a 2-window sliding window before and after the peak on April 3, 2020. Predicted doubling times each day are *T* = ln 2/ln *r*. *r* < 1 is the condition for exponential decay. We can then speak of a “halving-time.”

Day (*n*)	*r*_*n*_	Predicted *T*_*n*_ (days)
March 25	1.22	3.5
March 26	1.1	7.2
March 27	1.08	9
March 29	1.03	
March 30	1.08	
March 31	1.01	69
April 1	1.02	
April 2	1.01	69
April 3	0.98 (peaked)	
April 4	0.97	

**TABLE IV.** Germany’s daily infection rates near the peak on April 6, 2020.

Day (*n*)	*r*_*n*_	Predicted *T*_*n*_ (days)
April 2	1.07	10.2
April 3	1.04	
April 4	1.03	23.3
April 5	1.04	18
April 6	0.96 (peaked)	

**TABLE V.** The daily infection rate in Italy is stuck and is approximately linear with *T* large.

Day (*n*)	*r*_*n*_	Predicted *T*_*n*_ (days)
April 5	1.03	23.3
April 6	1.02	
April 7	1.01	69
April 8	1.02	

**TABLE VI.** The unfavorable daily infection rate in the UK.

Day (*n*)	*r*_*n*_	Predicted *T*_*n*_ (days)
April 5	1.08	9
April 6	1.06	12
April 7	1.09	8
April 8	1.06	12
April 9	1.13	6

**TABLE VII.** The unfavorable daily infection rate in the USA while Austria, Germany, and some other countries had already peaked.

Day (*n*)	*r*_*n*_	Predicted *T*_*n*_ (days)
April 4	1.07	10
April 5	1.09	8
April 6	1.085	
April 7	1.07	10
April 8	1.07	
April 9	1.07	10
April 10		

The observed doubling time *N*_*n*_ is read directly from the data with a sliding window. One starts at day *n* in the data and slides the window forward until *I*(*n* + *N*_*n*_) = 2*I*(*n*). This method is limited because when you reach day *n* where *N*_*n*_ is larger than the number of days left in the dataset, then you must stop. This is a useful method so long as you are not at that limit. The resulting effective infection rate is then *r*(*n*, 1) = 2^1/*Nn*^ and depends on *n*. We illustrate this method in Table I, e.g., from March 27 to March 31 in the USA, the doubling time is 5.5 days. From March 2 to March 27, the doubling time increased in jumps from 2 to 5 days. One can also start at the last data point and read backward until the infections halve.

When in a decay of infections, *r* < 1, we can speak of the halving time *N* = −ln 2/ln *r*, satisfying *I*(*n*) = *I*(1)/2.

Before the March 16, 2020 lockdowns in Austria, Germany, and the USA, the respective doubling times were 2–3 days, 2.5–3 days, and 2.5 days for the three doubling intervals immediately preceding the lockdowns (roughly March 7, 2020–March 16, 2020). Table I gives the results that follow from reading the data.

Figures 1–5 show the differing effects of social distancing in reducing the infection rates in five different countries. The lockdown in Austria was strict, well obeyed, and successful (I was there the entire time and experienced it). Germany had a somewhat less efficient lockdown than Austria. The US and the UK first considered a free market response (no lockdown at all) and never locked down uniformly. I returned to the USA on May 8 and observed the behavior in Houston: most shoppers wore masks, with some few maskless people in grocery stores, clothing stores, and pharmacies. Masks were uniformly worn in a grocery store or pharmacy in the Austrian village where I lived. Italy did not react fast enough and then finally imposed a very severe lockdown that lasted long. The situation in Italy differed from that in Austria and Germany due to the explosion of cases in Bergamo where the hospitals and medical staff were severely overloaded (as later in NY City), and due to the lack of travel restrictions early on that could have limited the spread of the disease. In Italy, people were confined to their houses, which is hard to accept. In Austria and Germany, people went for daily walks. In Austria, we sometimes walked for hours outside the village.

There is no guarantee that a long predicted doubling time will be reached. Using the unrealistic but simple plateauing model where *r*_*n*_ = 1 + ε/*n*, we get *T*_*n*_ = ln 2/(ln(1 + ε/*n*) ≈ *n* ln 2/ε ≫ *n* for *n* large and ε small. Real data might peak and decay before such a limit is reached. This is to illustrate that the predicted doubling time is a simple and pretty good data-based forecast, it need not be what we actually observe after a time *T*_*n*_, e.g., the day before Germany peaked, *r*_*n*_ = 1.04 and *T*_*n*_ = 18. There was nothing in the active infections to suggest that that Germany would peak on April 6, 2020 and then decay on the next day, although I am sure that people who use technical analysis in the stock market could also find equally meaningless patterns in the covid data.

The active infections increased steadily in the UK through May 10. After that worldometer removed the data on active infections from their website. Deaths and total infections are still reported but without recoveries, which were never listed. Without recovery data, the active infections cannot be calculated (see Sec. III).

Finally, we compare the observed doubling time *N*_*n*_ with the daily predicted doubling time *T*_*n*_ = ln 2/ln *r*_*n*_ but leaving off the subscript *n*. The latter will provide a fairly decent prediction if and only if the daily rate *r*_*n*_ does not vary very much over the doubling time *T*_*n*_ that is predicted.

## III. SOCIAL DISTANCING VS RECOVERIES IN THE DATA

In what follows:

*n* = index labeling day *n*
*P*(*n*) = uninfected population on day *n* (not immune)
*I*(*n*) = total No. active infections on day *n*
*R*(*n*) = No. recoveries on day *n* (immune)
*D*(*n*) = total No. deaths on day *n*

Infections are reported on several daily data websites like worldometer^2^ and Johns Hopkins.^5^ There are total infections *I*_*T*_ where

$$I_{T}=I+D+R.$$ (11)

We will focus on active infections *I*(*n*), the source of new infections, assuming that dead and recovered patients cannot transmit the disease (to zeroth order, at least).

Total infections include active infections, recoveries, and deaths *I*_*T*_ = *I* + *R* + *D* so Δ*R* = Δ*I*_*T*_ − Δ*I* − Δ*D*. The world has *P*_*T*_ ≈ 7.6 × 10^9^ people with 81 × 10^6^ births/yr, and because the world population change is relatively small over a few weeks or a month, we can take *P*_*T*_ ≈ *constant* = *P* + *I* + *R*. This is the number conservation law,

ΔP+ΔI+ΔR=0, (12a)

where Δ*P* < 0 is the daily change in the uninfected, not-immune part of the population. We can apply (12a) to one country so long as deaths are small compared with recoveries. If deaths would rise to the order of magnitude as recoveries, then the required conservation law would be

ΔP+ΔI+ΔR+ΔD=0. (12b)

An infection peak occurs on day *n* when, on day *n* − 1, we find −Δ*P* > Δ*R* (Δ*I* = −Δ*P* − Δ*R* > 0), and then on day *n*, we find −Δ*P* < Δ*R* (Δ*I* = −Δ*P* − Δ*R* < 0). Comparison of the rates (or doubling times) for Austria, Germany, and Finland just a few days before their respective peaks shows that the peaks cannot be forecast, especially when compared with the US and UK data. Social distancing and vaccines reduce Δ*P* while recoveries are increased by a good healthcare system, a good immune system, or both. We will see the competing effects of both terms in the graphs. We could stop here, but we will additionally formulate these conditions using the standard chemical reaction kinetics with (12a).

Normally, chemical kinetics is formulated with differential equations because the reaction processes are fast on the time scale of one second. Our processes change on a time scale of the order of a day so that our kinetic equations are discrete. With concentrations of two species *n*_1_, *n*_2_ with reaction rate *k*, then *n*_1_ + *n*_2_ = *n* = *constant* so that Δ*n*_1_ + Δ*n*_2_ = 0. Assuming independence of the concentrations, then Δ*n*_1_ = *kn*_1_*n*_2_ = −Δ*n*_1_. The other assumption in chemical kinetics is that the populations are uniformly distributed spatially. With three different populations, it is similar unless one population does not react but simply grows directly from either population, say population 2. In that case, we have the number (or mass) conservation law as

$$n_{1}+n_{2}+n_{3}=constant$$ (13)

so that

$$\Delta n_{1}+\Delta n_{2}+\Delta n_{3}=0.$$ (14)

If populations 1 and 2 react while

$$\Delta n_{3}=mn_{2},$$ (15)

then

$$\Delta n_{1}=-kn_{1}n_{2}=-\Delta n_{2}-\Delta n_{3}$$ (16)

and so

$$\Delta n_{2}=kn_{1}n_{2}-mn_{2}.$$ (17)

In the chemical kinetics of binding of ions in an electrolyte^6^ to form neutral atoms, we would have *n* = *n*_1_ = *n*_2_ while Δ*n*_3_ = −Δ*n* = *kn*^2^ − *pn*_3_, where *k* is the association/binding rate while *p* denotes the dissociation/unbinding rate.

In our population notation defined above, we have

$$\begin{array}{c} \Delta P\left(n\right)=P\left(n+1\right)-P\left(n\right)=-b_{n}P\left(n\right)I\left(n\right),\\ \Delta I\left(n\right)=I\left(n+1\right)-I\left(n\right)=\left[b_{n}P\left(n\right)-g_{n}\right]I\left(n\right),\\ \Delta R\left(n\right)=R\left(n+1\right)-R\left(n\right)=g_{n}I\left(n\right). \end{array}$$ (18)

The only assumption is that the populations *P* and *I* are independent. A nonequilibrium thermodynamic application of the kinetic equations in continuous time to weak electrolytes is given in Ref. 6. When iterated globally from fixed initial conditions, our equations are called an SIR model.^7^ We do not present an SIR model here: we simply use the standard set of kinetic equations to organize the yesterday-to-today data. Epidemic and pandemic data rule out SIR model predictions with their fixed initial conditions.

In particular, we can extract the rate *r*_*n*_ = 1 + *b*_*n*_*P* − *g*_*n*_ daily. We obtain the recovery coefficient

$$g_{n}=\frac{\Delta I_{T}\left(n\right)-\Delta D\left(n\right)}{I\left(n\right)}-\left(r_{n}-1\right)$$ (19)

from (5). If we know the data for days *n* − 1 and *n*, then we can describe that transition. Were the rate coefficients constants fixed by thermal equilibrium (or by any equilibrium or steady state), the equations would have predictive power. However, unlike chemical kinetics, there is no equilibrium condition; the rate coefficients jump daily. Any discrete time SIR or SEIR model using the initial conditions is immediately falsified by the data. Instead, feeding the observed pandemic rates into the kinetic equations allows us to understand the effects of social distancing and recoveries.

The variable rate coefficient *b*_*n*_*P* reflects the effect of social distancing (or lack of same), while *g*_*n*_ is the variable recovery rate. A reduction in *b*_*n*_*P* early in the data (before recoveries become significant) tells that social distancing is working. The condition for peaking is *b*_*n*−1_*P* > *g*_*n*−1_ with *b*_*n*_*P* < *g*_*n*_. A condition to be near a possible peak is *r*_*n*_ ≈ 1, but being near the peak does not imply crossing it: we still need a jump that gives us *g*_*n*_ > *b*_*n*_*P*. You cannot get over the hump without feeding enough immune people into the population. Without recoveries (without immunity developed), the population simply dies out very, very slowly with *r*_*n*_ approaching unity from above. Therefore, a small positive growth rate *r*_*n*_ can lead either to (i) a continued epidemic with a long doubling time (*r*_*n*_ > 1 with the difference *r*_*n*_ − 1 small and positive, flattening without peaking) or (ii) a sudden jump on a single day from *r*_*n*−1_ > 1 to exponential decay (*r*_*n*_ < 1). Early in a contagion, the rate r is only reduced by social distancing. The daily rate coefficient separates these two important effects. As Onsager once commented, a good theory helps you to understand the data. On that count, we have a good theory.

## IV. FLATTENING AND/OR PEAKING

A contagion with a long doubling time can be approximated over shorter times by linear growth. If

$$r^{n}={\left(1+\varepsilon \right)}^{n}$$ (20)

with ε ≪ 1, then

$$\frac{I\left(n\right)}{I\left(1\right)}={\left(1+\varepsilon \right)}^{n}\approx 1+n\varepsilon .$$ (21)

This approximation may be seen in the data for a range of days Δ*n* ≪ *N*_*n*_. Approximately linear data over a few days do not reflect flattening, they merely reflect a longer doubling time. By flattening, we mean a definite slope reduction over several days. This can only be seen after it has occurred, flattening is not predictable. The often-read news statements that “the data seem to be flattening” or “the data are expected to flatten” are meaningless.

We will use the daily growth rate r to obtain the predicted daily doubling time *T* = ln 2/ln *r* for the most recent data. It is instructive to see how the peaks were approached in Austria and Germany.

The daily rate predicts a doubling time *T*_*n*_ = ln 2/ln *r*_*n*_. However, that doubling time will be reflected later in the data only if the data should become approximately linear on a time scale that is large compared with the doubling time.

The data for Austria approached the peak linearly and peaked on April 3, 2020. A rough estimate for March 12, 2020 gives *b*_*n*_*P* ≈ *r*_*n*_ − 1 ≈ 0.34 when *g*_*n*_ ≈ 0. However, while *P*_*n*_ ≈ *P* is constant, *b*_*n*_ changes with *n* due to social distancing. We next use the daily data to find

$$b_{n}P\approx \left(\Delta I_{T}\left(n\right)-\Delta D\left(n\right)\right)/I\left(n\right)$$ (22)

and

$$g_{n}\approx b_{n}P-\left(r_{n}-1\right).$$ (23)

On April 2, 2020, we find *b*_*n*_*P* = 0.025 and *r*_*n*_ − 1 = 0.01 or *g*_*n*_ = 0.015. On the next day, *g*_*n*_ > *b*_*n*_*P and r*_*n*_ < 0.

According to the data, Austria had a most effective lockdown: it peaked first and has had the fastest recovery (infection decay rate). Therefore, we should pay attention to the effect of lockdown for that case. The initial doubling time on February 29 was *N* = 3 days with *I* = 10 active infections. Austria peaked 44 days later on April 3, 2020 with *I* = 9334. Recoveries have little effect on r until distancing reduces the rate *b*_*n*_*P*, and *b*_*n*_*P* remains roughly constant without social distancing. Therefore, without social distancing, we could have expected *I* = 9334 active infections only 29 days later on March 29, 2020 (instead of the *I* = 8223 reported), and on April 3, 2020, we should have seen *I* = 29 702, three times the number reported under lockdown. Austria had a model lockdown.

Lockdowns in both Italy and Germany (less efficient but imposed at the same time) reduced the doubling rates in those countries significantly.

Germany peaked on April 6, 2020 (as did Iceland and Taiwan). We get *b*_*n*_*P* = 0.34 and *r*_*n*_ − 1 = 0.04 from the early data near March 16, 2020. From April 3, 2020 to April 5, 2020, *b*_*n*_*P* = 0.04 while *r*_*n*_ − 1 = 0.04 and *g*_*n*_ ≈ 0, so social distancing brought Germany to the peak, but the transition to *r*_*n*_ < 0 on April 6, 2020 was caused by recoveries.

When Italy finally locked down on March 9, 2020, there were already 4000 active infections (compared with 60 in Austria and 650 in Germany on March 16, 2020). To see in detail why Italy did not peak by April 6, 2020, we compare *b*_*n*_*P* with *g*_*n*_. On March 6, when *g*_*n*_ ≈ 0, we get *b*_*n*_*P* ≈ *r* − 1 = 0.28. On April 6, 2020, we find *b*_*n*_*P* = 0.04 and *g*_*n*_ = 0.03. There has been no jump to *r*_*n*_ < 1. Instead, Italy was in a long, slow increase in infections with *T* = 69 days. The daily rate was stuck at *r*_*n*_ ≈ 0.02 because the recovery rate coefficient *g*_*n*_ remained too small; peaking and decay are caused by recoveries, deaths do not contribute. Social distancing has been very strict in Italy, but the doctors and hospitals were overloaded and had to turn people away to die. The police and army were used to enforce stay at home orders in Italy; this would not have worked in the UK and the USA.

For the UK, we get from March 16, 2020 that *b*_*n*_*P* = *r*_*n*_ − 1 ≈ 0.27 (reflecting ineffective social distancing) but on April 9, 2020 *g*_*n*_ ≈ 0.001. On April 9, 2020, the UK is in the pandemic with a doubling time of 9 days with no peak in sight.

At lockdown on March 16, 2020, the USA had nearly half the infections of Germany. On March 16, we have *b*_*n*_*P* ≈ *r*_*n*_ − 1= 0.39 while on April 9, 2020, *b*_*n*_*P* ≈ 0.14 and *g*_*n*_ ≈ 0.004. Social distancing has been ineffective, and the recovery rate is much too low to compete with the much too large rate of new infections. Political policy is reflected in the covid-19 numbers.

Scandinavia presents a very good case for lockdown study because the five Scandinavian countries are culturally very similar with similar health systems and governments, even if Finland (and Estonia) has a non-Nordic ethnic origin. Iceland, Finland, and Denmark all peaked before or by mid-April, whereas the active infections were still growing in Norway and Sweden on April 27, 2020. Sweden did not lockdown. The death rate has been 15/100, twice as high as in the USA. Finland oscillated with large magnitude about *r* ≈ 1 from April 16, 2020 through May 10, 2020.

It is clear that strong regulations imposed early have worked very well in reducing the pandemic in Austria and Germany. It is equally clear that waiting too late and/or imposing half-measures half-heartedly does not work well (UK, USA). Next, consider the data provided by China.

The day-to-day data for China are smooth compared with all of the other data discussed here. The histogram in Fig. 6 was later generated by a computer program from fixed initial conditions (see Figs. 1 and 4 in Ref. 8). That model used the memory of three past rates [Eqs. (12) and (13) in Ref. 8 with J = K = 3, pg. 9 of Ref. 8], which is the same as using four initial conditions in the iterated maps of the model (see also the discussion of Ref. 9 in Sec. V). It would be interesting if the authors of Ref. 8 would apply their model to the complete data for Finland, Germany, Denmark, the USA, Cuba, and Taiwan, where the daily infections often change relatively abruptly. We could perhaps learn something useful from those results.

## V. COMMENTS ON MODEL CALCULATIONS

There are very many papers by groups attempting to model infection growth in a way that will convince other readers and grant agencies. We focus here only on two, the first because it is mentioned so often in the US news, and the second because it is easy to understand and use the model: as with this paper, a hand calculator along with the daily data are enough.

We do not have access to the IHME model^10^ whose predictions have been reported and revised all too often in the news media. A description of the model is provided in Ref. 11, where the authors state that data from China are used by IHME to inform their forecasts. Social distancing, lockdowns, and recoveries vary significantly from country to country, and these are the factors that change the daily rates. The data from one country do not inform us of the data from another; Austria and Germany have performed somewhat similarly, but compare Norway and Denmark to see a great contrast. The IHME model predicted on April 20, 2020 that the US deaths would peak on April 21, 2020 and then decay. Instead, on April 20, 2020 *D* = 42 853 and on April 26, 2020, *D* = 55 413 with a predicted doubling time *T* = 34 days. Neither peaking nor even flattening were indicated by the data when the prediction was made. Long range predictions are impossible, but this did not prevent IHME from predicting that *D* = 67 640 will remain constant from July 4, 2020 onward. The reality is that *D* = 55 413 on April 26, 2020 with *T* ≈ 146 days. Better predictions can be made with one simple equation using only a hand calculator.

Consider next a simple model where all calculations can be made by hand. The model^9^ replaces the observed daily rate by an average of daily rates over the preceding *m* + 1 days (see also Ref. 8). The authors state that we need to keep the infection growth rate below 5% (*r* − 1 < 0.05) in order to plateau. However, the data do not reflect “a highly nonlinear process” as is claimed,^9^ and we have seen that the data reflect an iterated linear map. In the model, *x*_*n*_ is supposed to represent the active infections *I*(*n*) on day *n*. The model ansatz is based on storing *m* + 1 states of initial data as memory,^9^

$$\frac{x_{n+1}}{x_{n}}=\frac{\frac{x_{n-1}}{x_{n-2}}+\frac{x_{n-2}}{x_{n-3}}+\cdots +\frac{x_{n-m}}{x_{n-m-1}}}{m},$$ (24)

where the rhs replaces the daily rate *r*_*n*_ = *I*(*n*)/*I*(*n* − 1) by the quantity

$$R_{n}=\frac{\frac{x_{n-1}}{x_{n-2}}+\frac{x_{n-2}}{x_{n-3}}++\cdots +\frac{x_{n-m}}{x_{n-m-1}}}{m}=\frac{x_{n+1}}{x_{n}}$$ (25)

so that we are studying the terms *x*_*n*+1_/*x*_*n*_ = *R*_*n*_ rather than the observed infection rate data *I*(*n*)/*I*(*n* − 1) = *r*_*n*_. Rather than the single initial condition required to iterate map (5), one needs *m* + 1 initial conditions to iterate map (24). The initial conditions may or may not be taken from real data. Even if the *m* + 1 initial conditions are taken from real data, then the iterates for *n* ≥ *m* + 1 will not be the real data. In any case, can we find a peak and decay in the model’s future iterates? This has nothing to do with the data analysis or the epidemic prediction but it is an interesting mathematical game. With

$$\frac{x_{n+2}}{x_{n+1}}=\left(mR_{n}+\frac{x_{n}}{x_{n-1}}-\frac{x_{n-m}}{x_{n-m-1}}\right)/m,$$ (26)

a negative term appears. In the next iteration,

$$\frac{x_{n+3}}{x_{n+2}}=\frac{mR_{n}+\frac{x_{n+1}}{x_{n}}+\frac{x_{n}}{x_{n-1}}-\frac{x_{n+1-m}}{x_{n-m}}-\frac{x_{n-m}}{x_{n-m-1}}}{m},$$ (27)

we pick up two negative terms, and so on. The (*n* + *N*)th iterate has *N* − 1 differences in the rate. However, there is no such relationship between a past average and future rate jumps in real infection data. The difference/error in *r* − *R* will be magnified exponentially as maps (5) and (25) are iterated. If we use as initial data the eight infection rates from Germany for March 25, 2020 through April 1, 2020, where the data are only five days short of peaking on April 6, 2020, then we find from (26) and (27) that the predicted infection rates pass through the date April 6, 2020 at 1.05 and then increase to 1.06 on April 7. In the real data, *r* = 1.04 on April 25, 2020 but then *r* = 0.96 < 1 on April 6, 2020. In the model, the infections continue to increase without peaking. Germany and Austria peaked sharply without flattening. Real data may also flatten without peaking. Flattening should not be imagined as a precursor to peaking.

If the *m* + 1 initial conditions are chosen carefully so that the successive slopes are decreasing (as in Fig. 1 of Ref. 9 that looks nothing like the worldometer pandemic data), then the earlier negative terms may eventually win over the later positive ones and *R*_*n*_, causing peaking and decay. However, since the real, observed data do not obey (24), this ansatz unfortunately does not inform us about epidemics. We do not know any correct rule where the observed rate *r*_*n*_ is given by any combination of preceding rates *r*_*n*−2_, *r*_*n*−3_, …. Rule (25) does not generate the covid data, so the authors’ 5% rule^9^ follows from choosing initial conditions carefully in the numerical game, not from an epidemic data analysis. Presumably, one must choose initial data so that *R* < 1.05 in order to flatten and peak in the model. However, past initial conditions do not cause flattening or peaking of a real epidemic, only social distancing and recoveries can do that. There is a 1–2-week time lag between becoming infected and showing illness, but that is not the 1–2-week time lag in the model’s memory above. There is no way aside from a direct corona virus test to know who is infected until the person becomes ill.

Summarizing, one can choose initial conditions in the model that are favorable for smooth peaking and decay. In an epidemic, we have no control over initial conditions, the peaking is generally not smooth, and we must deal with bad initial conditions that evolve exponentially with *r* ≈ 1.2–1.3 and then find a way to bring down the infection rate. Unlike (24), we have on hand no mathematical rule that tells us how the rates *r*_*n*_ evolve. The infection rates are algorithmically complex:^4^ the shortest computer program that can generate any *n* daily rates is simply to write down the *n* observed rates on a given day. It is obvious that there can be no simple hidden rule to tell us the rates because the change in *r* depends on how people respond to social distancing, and we do not know that in advance (simply compare Austria, Germany, the USA, Iceland, Denmark, Thailand, and Finland, all of whom locked down to different degrees).

## VI. COVID EVOLUTION WITH RELAXED RESTRICTIONS

**FIG. 8.**
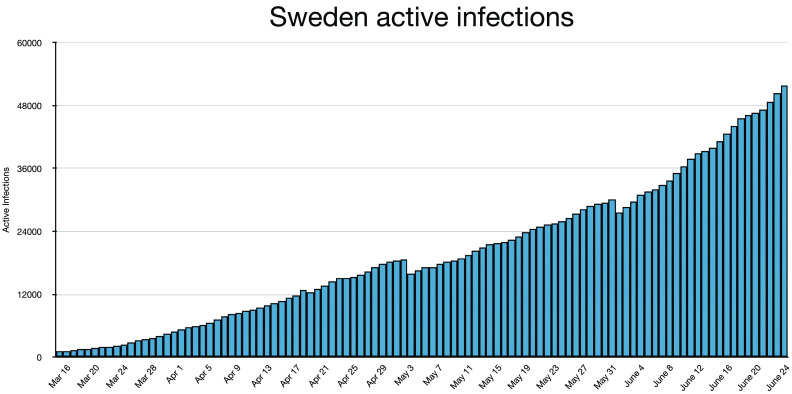
Active infections are shown for Sweden.

**FIG. 9.**
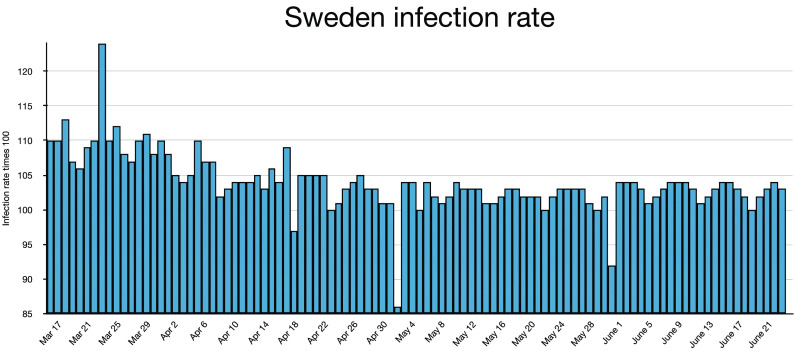
The infection rate is shown for Sweden, a land with relaxed social and travel restrictions.

We would like to know the natural, unchanged rate of infection for covid for normal social interactions. The Swedes have provided us information necessary to find a rate where freedom was largely unrestricted: Sweden has had no lockdown, has weak requirements for social distancing, and no requirement to wear masks. Schools were closed after 1 April but the social distancing requirements are very weak: up to 50 people were allowed in gatherings and amusement parks. As of 27 April, high schools and universities were closed, but nightclubs, restaurants, and bars remained open. Only nonessential travel from non-EU countries was finally banned. Sweden’s chief epidemiologist is reported to have asserted^12^ on 15 April that infections had plateaued and may have peaked. Nothing was further from the truth, as Figs. 8 and 9 show.

We can expect good statistics when the infections are high from a large number of collection sites. For Sweden with *P*_*T*_ = 10^7^, as of August 6, 2020 *I*_*T*_ = 81 500 with *D*/*I* = 0.085 (the US death rate is 0.071). We assume that the rate for Sweden should be typical for any western country with similar relaxed social interactions. An Asian country like Japan, with different daily habits and customs, might show a different rate under similar circumstances.

Sweden shows *I* ≈ 10 000 around April 10, 2020, Easter weekend. Beyond that date, the most probable rate is r ≈ 1.03–1.04; the effective rate for the 75 days from April 10 to June 25 is r_eff_ = 1.02. Thrice, the rate falls to *r* < 1 but immediately returns to *r* > 1. The infection rate is certainly small enough for peak and decay, but peak and decay never occur because, without lockdown, social distancing, and masks, the daily infections always outnumber the daily recoveries. This is a lesson for the world: if you let covid run unabated, then you can expect it to continue until the entire population is infected after a very long time. In the case of Sweden, we can then expect nearly a million deaths, or close to 10% of the population. At the current low infection rate (*r* = 1.001 on August 06, 2020), this should take about 13 years. Infecting 60% of the total population would take about 465 days. The rates dropped below 1.00 during July, the vacation month. The official policy of Sweden amounts to trying to plateau without ever peaking even if that is not the intention.

## VII. SUMMARY

Infection rates under complete or partial lockdown can be well defined to several decimal places but are not predictable in advance, they can only be discovered as they unfold daily. Modeling the rates artificially does not lead either to insight or to predictability. The reality of the unfolding reflects the complexity of the underlying process that determines the coefficients in the kinetic rate equations. The kinetic equations are simple but the process that determines the rates is algorithmically complex. Definitions of complexity are discussed in chap. 11 of Ref. 4. If we know the infections from today and yesterday, then we can use Eq. (5) to forecast the infections for a few days in advance. This is a useful forecast based on current data. More complicated computer models are neither more accurate nor more useful, they only cost money that could be better spent elsewhere.

We have quantified the effect of social distancing, and we can extract that effect from the daily data. We have defined flattening/plateauing and also peaking mathematically precisely. To predict plateauing, one would have to predict correctly the daily changes in social distancing. To predict peaking and decay, one would have to be able to predict the date on which new recoveries overtake new infections. The jump from growth to decay of infections is only possible if the rate at which immune people are fed back into the population is greater than the rate at which susceptible people become infected; both rates are extracted from the daily data by using number conservation for the populations with or without the kinetic equations.

The implications for public health are simple: first, we see that strictly obeyed lockdowns can work very well (Austria, Germany, Denmark, etc.) while half-hearted ones do not (USA and UK). Early response with closure of borders (Austria, Germany) beats late response with open borders and also beats, as in the case of Italy, a very severe lockdown imposed far too late with borders open too long. Recoveries are aided by a strong health system. The USA lags behind Europe in universal health care; in the USA, people with no health insurance cannot be turned away by hospitals but typically land at a county hospital rather than at a better equipped private one. Second, model building does not and cannot reflect real epidemic data because the only correct rates emerge day by day in the daily data, cannot be usefully forecast for more than a few days in advance, and cannot be forecast at all correctly near a peak. Significant differences in social distancing can be seen in the data during the decays: Austria required 2.5 weeks from lockdown to peak and then needed another 5 weeks to decay back to the level of lockdown. In Germany, the peak occurred 3 weeks after the lockdown but Germany needed nearly three months before the active infections fell from the peak to the level at lockdown. At lockdown, the infections in Germany were seven times higher than in Austria, although Ischgl, Austria, was closer to the proclaimed source in Bergamo, Italy. Social distancing in Italy after the peak was a failure: on July 7, nearly three months after the peak, there were 6000 more active infections than at lockdown on March 09, 2020. There was, therefore, no ground based on covid-19 infections for Austria and Germany to open their borders with Italy or the UK in June 2020. Finally, modeling does not help to reduce the spread of an epidemic and is not needed; lockdowns and social distancing do stop the exponential growth.

We cannot model infection rates but we can still say something useful. Consider the case of a market bubble before the liquidity dries up and the bubble has popped. There is no way to predict mathematically beforehand when the bubble will pop and the market will crash because, for one thing, the number of points representing a market crash are far too few for an ensemble analysis. In the absence of a mathematical prediction, there are still signs of an inflating bubble: there is always an unusual behavior like people mortgaging their houses to day-trade stocks as in 2000, and the buying and rapid resale of houses as if they would be stocks in 2007 (shadow banking,^4^ with the US dollar amounting six to seven times M3, was a sign of the bubble but few people knew about shadow banking until it was too late). Likewise, in a pandemic or epidemic, if a country locks down very efficiently including closing borders and preventing travel outside the home region, then we can expect that the exponential growth of the epidemic will peak and begin to decay within about 3 weeks. If strict social distancing continues after the peak, then the decay will also continue (in strict social distancing, there is no association of people from different homes). We have learned this from the examples of several countries and from the counter example of the USA, and no mathematical model is needed to predict that. Experience is adequate in this case, as in the case of market bubbles.

Cell biology is all about complexity. As Ivar Giaver once remarked about a cell biology text^13^ at a Geilo NATO-ASI, there are many facts therein but very few equations to describe them. Ivar also asserted that either we (physicists with our equations) are right or they are right, and if we are right, then we need to add equations to “Fat Alberts,” as Ref. 13 is known. The challenge is a good one but adding equations to cell biology is not simple. For example, the map (1) is simple but the rates that generate the time evolution cannot be known in advance of their occurrence. I end with the paraphrase of a comment on simplicity vs complexity by von Neumann:^14^ … it is characteristic of simple systems that it may be easier to predict their properties mathematically than to build them (Earth-Sun motions, e.g.), … for complex systems it is harder mathematically to predict their behavior than to produce or observe the object (e.g., viral and bacterial mutations).

## Data Availability

The data used herein were taken from worldometer.^1^
